# Association between contact with mental health and substance use services and reincarceration after release from prison

**DOI:** 10.1371/journal.pone.0272870

**Published:** 2022-09-07

**Authors:** Emma G. Thomas, Matthew J. Spittal, Faye S. Taxman, Cheneal Puljević, Edward B. Heffernan, Stuart A. Kinner

**Affiliations:** 1 RAND Corporation, Santa Monica, California, United States of America; 2 Centre for Mental Health, Melbourne School of Population and Global Health, The University of Melbourne, Melbourne, Australia; 3 Criminology, Law & Society, College of Humanities and Social Sciences, George Mason University, Fairfax, Virginia, United States of America; 4 School of Public Health, The University of Queensland, Brisbane, Australia; 5 Centre for Health Services Research, The University of Queensland, Brisbane, Australia; 6 Queensland Forensic Mental Health Service, Brisbane, Australia; 7 Justice Health Unit, Melbourne School of Population and Global Health, The University of Melbourne, Melbourne, Australia; 8 Centre for Adolescent Health, Murdoch Children’s Research Institute, Melbourne, Australia; 9 School of Population Health, Curtin University, Perth, Australia; 10 Griffith Criminology Institute, Griffith University, Brisbane, Australia; University of North Carolina at Chapel Hill, UNITED STATES

## Abstract

**Objective:**

People released from prison who experience mental health and substance use problems are at high risk of reincarceration. This study aimed to examine the association between contact with mental health and substance use treatment services, and reincarceration, among adults released from prison.

**Methods:**

Pre-release survey data from 1,115 adults released from prisons in Queensland, Australia were linked with administrative health and correctional records covering a median of 787 days post-release. We constructed marginal structural Cox proportional hazards models, adjusting for pre-release variables and time-varying indicators of emergent mental health and substance use problems, to examine the association between contact with mental health and substance use treatment services, and reincarceration.

**Results:**

The adjusted hazard ratio (AHR) for reincarceration associated with mental health service contact was 1.76 (95%CI 1.23,2.51). Among those not on parole following release, the AHR for reincarceration associated with substance use treatment service contact was 3.16 (95%CI 2.09,4.77); we found no evidence for an association among those who were released on parole (AHR = 1.07; 95%CI 0.80,1.43).

**Conclusions:**

Although we cannot eliminate the possibility of residual confounding, our findings suggest that infrequent or unsustained contact with community-based mental health and substance use treatment services is not protective against reincarceration, and may even be iatrogenic. Increased investment in high-quality and timely behavioural health services for people released from prison may simultaneously improve health outcomes, and reduce reincarceration.

## Introduction

Globally around 11 million people are incarcerated on any given day, and the world prison population is growing at a rate in excess of general population growth [1]. People who experience incarceration are at elevated risk of complex health problems, including substance dependence and other psychiatric disorders, compared to the general population [2, 3]. After release from prison, these individuals also experience high rates of poor health outcomes including nonfatal drug overdose [4, 5], self-harm [6], and preventable death due to drug overdose and suicide [7–10]. Another persistent challenge facing people released from prison is the risk of reincarceration, which remains normative in most countries where data are available [11]. This challenge is compounded for those with unresolved health problems, particularly substance dependence and other psychiatric disorders [12–14]. However, despite a widespread belief that access to substance use treatment and community mental health services after release from prison can reduce recidivism rates [15], strong empirical evidence for this association remains elusive. We are aware of only three studies that have investigated the association between use of community-based substance use or mental health services (as a standalone intervention) and return to custody. In two studies, the authors reported that receipt of mental health and substance use services was associated with a decrease in recidivism [16, 17]. However, both studies were retrospective, relied exclusively on administrative data, and were unable to comprehensively adjust for confounding due to the limitations of their administrative data. The third study, also using retrospective linkage of administrative data, found that individuals with serious mental illness released from prison on parole, who received timely mental health services post-release, were more likely than those who did not receive timely treatment, to experience re-incarceration [18]. The authors noted that this association was consistent with findings from previous studies of multi-component interventions showing that post-release mental health treatment increased the likelihood of technical parole or probation violations being detected, thus increasing risk of re-incarceration [19–22].

Given the high rates of mental illness and substance use disorders among people who experience incarceration (including those undiagnosed or experiencing symptoms not meeting a clinical threshold for diagnosis) [2, 3, 16], that risk of experiencing mental illness and substance use disorders is elevated after release from prison [4–6], and that accessing mental health and/or substance use services following release from prison may be a mandatory parole condition for some, it is important to understand the impact of access to mental health and substance use services among the population of people released from prisons. As such, in this prospective cohort study of formerly incarcerated men and women, we address two related questions: 1) what is the association between receipt of mental health and substance use services and risk of return to custody; and 2) does the timing and/or continuity of service contacts modify any observed association with return to custody?

## Methods

We conducted a cohort study of 1,325 sentenced adults released from seven prisons in Queensland, Australia, recruited between August 2008 and July 2010. Data were originally gathered as part of a randomised controlled trial (RCT) of a brief case management intervention. Once the trial was complete, additional data linkage was undertaken to treat the sample as a cohort study. We linked data from interviews conducted prior to release from prison with electronic health and criminal justice records up to four years post-release. All data included in this study were collected between 1 August 2008 and 31 July 2012. Ethics approval for this study was granted by The University of Queensland Behavioural and Social Sciences Ethical Review Committee, and the Queensland Health Human Research Ethics Committee. All participants provided written informed consent. Linkage with administrative health records was approved with a waiver of consent under the Queensland Public Health Act (2005).

### Data sources and linkage

The original RCT was of a case management intervention, described in more detail elsewhere [23]. Briefly, eligible participants were sentenced adults within six weeks of expected release from prison (full-time or on parole) at baseline interview. On demographic and criminal justice variables, the cohort was representative of adults released from Queensland prisons over the same time period, except that women were intentionally over-sampled [23]. Baseline interviews were conducted in person by trained researchers using a structured questionnaire. Upon release, participants in the intervention group received a personalised booklet summarising their health status and identifying relevant health services in the community. Trained health workers attempted to contact members of the intervention group by telephone weekly for the first four weeks after release, in an effort to facilitate health service access. Both trial arms were included in the present study, with control for randomisation to the intervention arm.

Dates of release and re-incarceration during follow-up were obtained from Queensland Corrective Services (QCS). Dates of death during follow-up were obtained from the National Death Index (NDI). We obtained data on community mental health and substance use service use from the state-wide Consumer Integrated Mental Health Application (CIMHA) and Alcohol, Tobacco and Other Drugs Services (ATODS) databases, both operated by Queensland Health. Morbidity data came from the Queensland Hospital Admitted Patient Data Collection (QHAPDC), the Emergency Department Information System (EDIS), and Queensland Ambulance Service (QAS) patient records. Correctional (QCS) data were linked deterministically using unique prisoner identification numbers. The NDI, CIMHA, ATODS, QHAPDC, and EDIS databases were linked on name, sex, date of birth, address, and any aliases [24] using a probabilistic linkage methodology with a previously estimated false linkage rate of less than 0.1% [25]. QAS data were linked by structured, manual interrogation of electronic ambulance records.

### Outcome and exposure

The outcome of interest was time from prison release until first reincarceration (if any) in Queensland, including parole violations or new offences. The exposures of interest were contact with community mental health and substance use treatment services after release from prison, treated as time-varying covariates (see Statistical Analyses for more details).

### Covariates

Covariates selected for inclusion in statistical models were considered to be potential confounders of the relationship between the exposures and the outcome. Here, we describe key mental health- and substance use-related confounders, and briefly outline other covariates included. Due to the large number of covariates, more detailed definitions are provided in S1 and S2 Tables.

#### Time-constant covariates

The following variables were treated as time-constant in all statistical models. Time-constant variables were determined by self-report in the baseline survey unless otherwise indicated. All variables derived from the baseline survey were included in models as continuous covariates unless otherwise stated.

We considered lifetime indicators of poor mental health, and mental health status at baseline. Lifetime indicators included any diagnosis with an anxiety disorder, a mood disorder, or schizophrenia. Baseline mental health variables included prescription for any central nervous system (CNS) medication according to prison medical records, intellectual disability assessed using the Hayes Ability Screening Index (HASI [26]), Kessler Psychological Distress Scale [K10; 27] score, and mental health-related functioning as measured by the SF-36 mental health component summary score [28].

We included only lifetime and pre-incarceration indicators of substance use problems, given that many people cease or reduce their use of illicit substances during incarceration [29]. Lifetime variables included history of injection drug use, history of sharing injecting equipment, history of injecting in prison, and history of overdose. Risk of alcohol-related harm in the six months prior to incarceration was measured using the Alcohol Use Disorders Identification Test [AUDIT; 30]. Risk of harm due to illicit drug use in the three months prior to incarceration was measured using the Alcohol, Smoking and Substance Involvement Screening Test [ASSIST; 31], with separate variables included for harm associated with each of cannabis, amphetamines, and heroin or other opiates.

Demographic baseline covariates included age, sex, Indigenous status, and marital status at baseline. Criminal justice covariates derived from correctional records included parole status at release, history of past incarceration as an adult, drug-related sentence, violent offence, and the QCS Risk of Reoffending (ROR) Score. History of juvenile detention was measured by self-report. Indicators of socioeconomic status included income, education, employment, and housing prior to incarceration. We also included measures of social support at baseline interview, and the geographic remoteness of the participant’s most likely residence after release. As these data were originally from an RCT, we included randomisation group as a covariate. Because the intervention was administered at release, and was low-intensity and time-limited (up to four weeks post-release), randomisation group was treated as a time-constant baseline covariate.

#### Time-varying covariates

We also controlled for time-varying indicators of emergent mental health and substance use problems after release from prison. These were defined as acute health service contacts for mental health or substance use problems, ascertained from linked health records. Three types of acute health service contact were considered, leading to six time-varying covariates overall: hospitalisations, ED presentations, and ambulance attendances, for mental health and/or substance use problems. The reason for each contact was ascertained from the ICD-10-AM code of the primary diagnosis for hospitalisations and ED presentations, and from case definitions provided by paramedics for ambulance attendances. Each type of acute health service contact was treated as a time-varying indicator variable representing any contact with that service in the past 30 days. This time window was chosen based on expert knowledge as the likely period during which any increased effects of an acute mental health or substance use-related episode might be observed.

### Statistical analyses

We used Cox proportional hazards regression to assess the association between contact with mental health or substance use services and time to first reincarceration. Time-to-event data were censored on 31 July 2012 (end of the study period) for participants who had not returned to custody, or at death for those who died before this date. We constructed time-varying indicator variables representing the period before and after first contact (if any) with each of mental health and substance use treatment services. In preliminary analyses we tested if the effect of substance use treatment services differed by parole status at release from prison and, on the basis of a significant interaction (p<0.001) and because some participants may have accessed substance use treatment services due to a mandatory parole condition, stratified our results by parole status. No such interaction existed for community mental health services (p = 0.197), and hence the results for this exposure are reported for supervised and unsupervised participants combined.

We approached model building in three steps. In Model 1 we fit unadjusted Cox models. In Model 2, to adjust for confounding by baseline factors, we refit the models controlling for all baseline variables. In Model 3, in addition to baseline variables, we adjusted for potential time-varying confounders observed after release from prison by fitting a marginal structural model via inverse probability of treatment weighting [IPTW; 32]. For the model examining mental health services, we adjusted for contact with substance use services and vice versa. We estimated subject-specific, time-varying IPTWs and incorporated these into the proportional hazards models, accounting for within-subject correlation when calculating standard errors [33]. Detailed methods for Model 3 are provided in S1 Text.

To assess the impact of timing and frequency of mental health and substance use service contact on the outcome, we performed two sets of secondary analyses. First, we divided the exposures into early versus later service initiation, using separate indicator variables if the first contact occurred early (<30 days after release) versus later (≥30 days after release). This one-month period is widely regarded as a critical time window during which people released from prison are especially vulnerable to substance-related harm [34], and there is evidence that contact with health services during the first month post-release is important to ensuring ongoing engagement with care [35]. Second, we divided the exposures into single service use versus repeat service use, with separate indicator variables representing the period between first and second service contact (if any), and the period following the second contact. In each case, we re-ran our models to examine any differential effects of these breakdowns of the exposure variables, and tested for differences in the log hazard ratios (HRs) using Wald tests. This analysis was performed on all observations where mental health service use was the exposure, and was restricted to people not on parole when substance use service contact was the exposure of interest.

Finally, to assess any possible bias produced by the inclusion of both trial arms from the original RCT in our study, we repeated all of the above analyses in only the control group. All analyses were performed in Stata 13.1.

## Results

Of 1,325 participants recruited at baseline, 1,307 (98.6%) had been released from prison at the time of administrative data extraction and were successfully linked to all administrative datasets. Of these, 1,115 participants (84.2% of the original cohort of 1,325) had data available on all covariates and were included in the final analyses. S1 Table shows the number with missing data for all variables. Most individual covariates were missing no more than 2.2% of values. The exception was the prescription CNS medications variable, which was missing for the 7.1% of participants who did not give consent for the researchers to access their prison medical records. All subsequent analyses were performed using the 1,115 complete cases.

The median age of participants at baseline was 33 years; 21% were female, and 23% identified as Indigenous (Table 1). Thirty-six percent of participants engaged in high-risk alcohol use in the six months prior to incarceration and 55% had a history of injecting illicit drugs. Twenty-six percent of participants reported high or very high psychological distress at baseline and 30% were being treated with at least one psychotropic medication. The median follow-up time was 787 days (IQR: 237, 1077) from release until re-incarceration, death or censoring. Fifty percent of participants returned to custody during the study period, and 60% were on parole at release.

**Table 1:** Baseline characteristics of participants (n = 1,115).

Variable	Value^b^
*Demographic*	
Age -- %	
18–24 years	24.3
25–39 years	53.0
40+ years	22.8
Female -- %	20.5
Indigenous -- %	23.0
Not married or de-facto -- %	66.4
Intervention arm, PASSPORTs -- %	50.4
*Criminal justice*	
Prior incarcerations (adult) -- %	67.2
Juvenile detention -- %	27.3
Drug-related sentence -- %	30.9
Violent offence -- %	52.1
On parole at release -- %	60.3
Risk of re-offending score—mean (SD)	11.2 (6.0)
*Mental health*	
CNS medications -- %	30.0
Mood disorder -- %	17.2
Anxiety disorder -- %	7.5
Schizophrenia -- %	3.8
High/very high psychological distress (K10) -- %	25.6
Low mental health functioning (SF-36)^b^ -- %	4.8
*Substance use*	
Ever overdosed -- %	23.3
Ever injected illicit drugs -- %	55.4
Ever shared injecting equipment -- %	27.3
Ever injected in prison -- %	20.9
High risk drinking^c^ (AUDIT) -- %	36.3
High risk cannabis use^d^ (ASSIST) -- %	46.2
High risk heroin use^d^ (ASSIST) -- %	17.6
High risk use of other opiates^d^ (ASSIST) -- %	21.0
High risk amphetamines use^d^ (ASSIST) -- %	38.4
*Social support*	
No visits past four weeks -- %	52.5
Low perceived social support (ESSI) -- %	18.7
*Socioeconomic*	
<10 years education -- %	42.5
Unstable housing^a^ -- %	20.8
Unemployed^a^ -- %	51.0
Income below poverty line^a^ -- %	46.2
Expected post-release postcode -- %	
Major city	73.2
Regional	24.5
Remote	2.3

^a^Prior to index incarceration

^b^Score two standard deviations below the mean

^c^In the six months prior to incarceration

^d^In the three months prior to incarceration

Table 2 summarises use of mental health and substance use treatment services after release from prison. Nineteen percent of participants had at least one community mental health service contact during the follow-up period. The median time until first contact was 170 days (185 days for those on parole, 159 days for those not on parole). Among those who contacted community mental health services, 36% had just one contact, 18% had two contacts, and 46% had three or more contacts. The majority of participants who contacted mental health services had at least one intake (57%) or assessment (54%) consultation; fewer than one in four received counselling/therapy or crisis management (see S3 Table).

**Table 2:** Summary of health service use for mental health and substance use problems during follow-up.

Health service	Any contact	Days until first contact for service users	Number of contacts for service users		
			Percent (number)		
	Percent (number)	Median (IQR)	1	2	3+
*Community mental health and substance use services*					
Mental health services	19.3 (215)	170 (29, 371)	35.8 (77)	18.1 (39)	46.1 (99)
Substance use services	24.1 (270)	130 (49, 356)	50.4 (136)	34.1 (92)	15.6 (42)
*Hospital admission*					
Mental health-related	3.9 (44)	307 (117, 507)	68.2 (30)	18.2 (8)	13.6 (6)
Substance use-related	4.2 (47)	261 (123, 571)	87.2 (41)	10.6 (5)	2.1 (1)
*Emergency department presentation*					
Mental health-related	5.3 (59)	253 (88, 489)	62.7 (37)	18.6 (11)	18.6 (11)
Substance use-related	6.4 (71)	256 (113, 563)	69.0 (49)	16.9 (12)	14.1 (10)
*Ambulance attendance*					
Mental health-related	4.5 (50)	395 (174, 718)	86.0 (43)	8.0 (4)	6.0 (3)
Substance use-related	5.3 (59)	244 (86, 554)	83.1 (49)	11.9 (7)	5.1 (3)

substance use = alcohol and other drug; IQR = interquartile range

Twenty four percent of participants accessed community substance use treatment services. The median time until first contact was 130 days (119 days for those on parole, 211 days for those not on parole). Fifty percent had just one contact with substance use treatment services, 34% had two contacts, and 16% had three or more contacts. The most common types of substance use treatment service accessed were assessment (36%) and counselling (34%).

Table 2 also details access to acute and tertiary services for mental health- and substance use-related reasons. Contact with these services for mental health or substance use reasons was rare (no one service was used by more than 6% of the sample) and, in the majority of cases, there was only one such contact.

### Association between mental health and substance use service contact and reincarceration

Table 3 presents the hazard ratios (HRs) for each main exposure, estimated by three different models: Model 1 (unadjusted), Model 2 (adjusted for baseline covariates), and Model 3 (adjusted for baseline covariates and post-release, time-varying covariates). We present only the HRs of interest here; complete results, including HRs for all covariates, are shown in S4 and S5 Tables.

**Table 3:** Effect of mental health and substance use treatment services on hazard of re-incarceration.

Model	Mental health services	Substance use services	
	All	Not on parole	On parole
	(n = 1,115)	(n = 442)	(n = 673)
	HR (95%CI)	HR (95%CI)	HR (95%CI)
Model 1^a^	2.57 (2.01, 3.29)	4.14 (2.92, 5.88)	1.49 (1.16, 1.92)
Model 2^b^	1.97 (1.43, 2.71)	2.99 (2.03, 4.40)	1.10 (0.82, 1.46)
Model 3^c^	1.76 (1.23, 2.51)	3.16 (2.09, 4.78)	1.07 (0.80, 1.43)

^a^Unadjusted

^b^Adjusted for pre-release covariates

^c^Adjusted for pre-release covariates and post-release (time-varying) covariates

Contact with mental health services after release from custody was associated with an increased risk of re-incarceration in both unadjusted and adjusted analyses. In unadjusted analyses, those who contacted mental health services had a 2.57-fold increase (95%CI 2.01, 3.29) in the hazard of return to custody, compared with those who did not contact mental health services. The magnitude of the HRs decreased towards 1 after controlling for baseline covariates (Model 2: HR = 1.97, 95%CI 1.43 to 2.71); controlling for time-varying confounders further attenuated the association (Model 3: HR = 1.76, 95%CI 1.23 to 2.59).

The results for the association between contact with substance use treatment services and risk of re-incarceration were more complex. Among those who were unsupervised on release (n = 442) contact with substance use treatment services was associated with an increased risk of return to custody. In Model 1, the HR was 4.14 (95%CI 2.92 to 3.29); adjustment for baseline covariates reduced this to 2.99 (95%CI 2.03 to 4.40) and, when further adjusted for the effect of time-varying confounders, the HR was 3.16 (95% CI 2.09, 4.78). Therefore, among people who were unsupervised upon prison release, the evidence suggested that contact with substance use treatment services was associated with an increased risk of re-incarceration. Among people who were released on parole, contact with substance use treatment services was associated with an increased risk of re-incarceration in unadjusted analyses (Model 1: HR = 1.49, 95%CI 1.16 to 1.92). However, after adjustment for pre-release covariates (Model 2: HR = 1.10, 95%CI 0.82 to 1.46) and time-varying confounders (Model 3: HR = 1.07, 95% CI 0.80, 1.43), there was no evidence of an association, although the 95%CI included a wide range of values including negative and positive associations.

**Fig 1:**
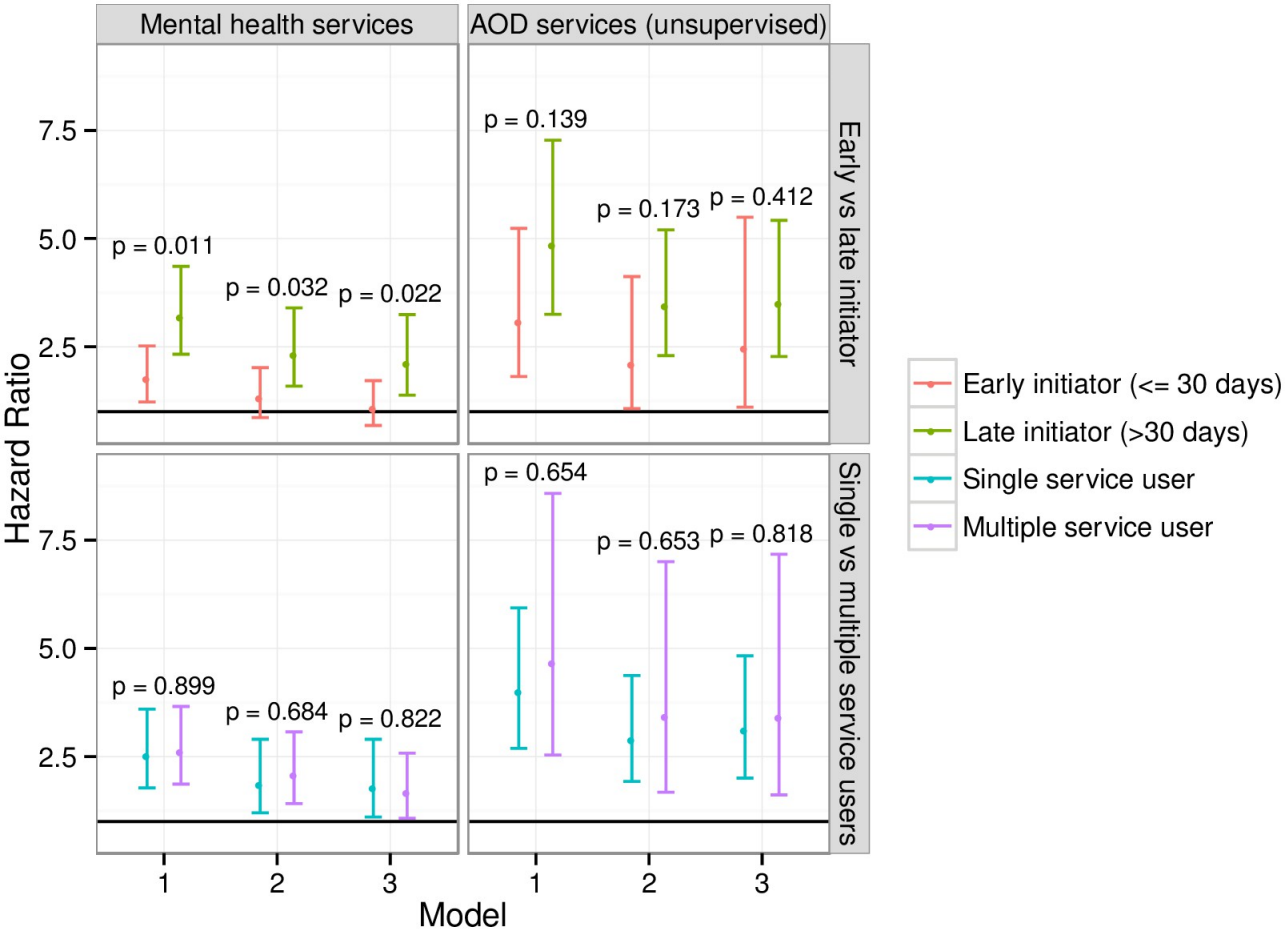
Results of secondary analyses (N = 1,116). The figure shows results from secondary analyses for our Cox proportional hazards models for time to re-incarceration. *Early initiators* are those who contacted services ≤30 days after release from index incarceration; *late initiators* first contacted services >30 days after release. *Single service users* are those who contacted services exactly once; *repeat service users* contacted services more than once. The p values shown are from Wald tests of the null hypothesis that the pairs of HRs for the breakdowns of each exposure are equal.

In secondary analyses we examined the effect of timing and frequency of mental health and substance use treatment service contact on risk of re-incarceration (Fig 1). In unadjusted analyses (Model 1), mental health service users who initiated contact within 30 days of release had a lower rate of return to custody than those who initiated later (p = 0.011), although both groups still had elevated re-incarceration rates compared with non-service users (HR = 1.76 for early initiators, 95%CI 1.23 to 2.52; HR = 3.19 for later initiators, 95%CI 2.33 to 4.36). This difference persisted after adjustment for baseline and time-varying covariates (p = 0.032 for Model 2, p = 0.020 for Model 3; see S6 Table).

Because we found no association between contact with substance use treatment and reincarceration for those released on parole, secondary analyses for substance use treatment services were restricted to those not on parole. We observed no evidence for a difference in the hazard of re-incarceration for unsupervised substance use treatment service users as a function of time to first contact. However, the risk of re-incarceration remained higher for service users than non-service users, particularly among those who were late initiators. We found no evidence for a difference in re-incarceration rates between those who contacted mental health or substance use treatment services only once versus repeatedly (see S7 Table).

Finally, since the cohort for this study was originally recruited for a RCT, we conducted sensitivity analyses in which the intervention arm of the trial was excluded and the main analyses were repeated in the control arm only. The results when restricted to the control arm did not differ qualitatively from the analysis in the entire cohort (see S8 Table). We thus conclude that the inclusion of the intervention group in our analysis is unlikely to have substantively altered our conclusions.

## Discussion

In this prospective study of adults released from prison, we found that contact with mental health and substance use treatment services was associated with increased risk of return to custody after controlling for pre-existing morbidity, known risk factors for recidivism, and evidence of emergent, acute mental health and substance use needs. Our findings are at odds with two previously published studies [16, 17] but align somewhat with a study that found increased likelihood of re-incarceration among a sample of people with serious mental illness who received timely mental health services following release from prison [18]. We believe the most plausible explanation for our results involves a combination of two factors.

First, participants in this study formed a heterogeneous group with a wide range of mental health and substance use service needs. Contact with these health services was likely to be indicative of emerging mental health or substance use problems, and these may not have been fully captured by the indicators of acute service need controlled for in this study. Therefore, despite careful adjustment for pre- and post-release confounders, we cannot exclude the possibility that our effect estimates are subject to residual confounding. To the extent that this is the case, our findings provide further evidence for the links between poor mental health, substance use problems, and reincarceration. For example, illicit substance use may have been detected through a mandated urine test a during parole appointment, resulting in a breach of parole conditions, or individuals may have been re-arrested for a new offence related to substance use (e.g., drug possession or public intoxication) or mental illness (e.g., disorderly behaviour).

Second, among participants in our study who did access mental health or substance use treatment services in the community, most had only one contact, often for assessment or administrative purposes. It seems unlikely that the level of service engagement seen here would be sufficient to address the complex needs of this population [36]. Thus, although some previous studies [16, 17] have reported reductions in recidivism associated with mental health and/or substance use programs targeted to people released from prison, we did not find similar evidence for the community-based services accessed by participants in our study. Instead, our findings suggest that delayed and infrequent engagement with behavioural health services after release from prison may be not only inadequate to prevent reincarceration, but potentially iatrogenic.

In secondary analyses we found that the positive association between mental health service contact and reincarceration was attenuated for those whose first service contact was within 30 days of release from prison. A possible explanation is that those who contacted services early had been referred to care due to pre-existing problems, while those who presented later were more likely to have experienced acute, emergent problems. A previous Australian study found that contacting primary care within 30 days of release from prison was associated with greater subsequent engagement with health services [35], suggesting a critical role for both pre-release referral and timely post-release case management to facilitate continuous engagement with care. Although we do not have any data indicating whether behavioural health service use in this cohort was court ordered, our stratification by timing of initiation (Fig 1) suggests that court-ordered service use, when initiated early enough, may not have the same iatrogenic effect observed among later initiators.

Whereas the association between mental health service contact and reincarceration was the same irrespective of parole status, the association between substance use treatment service contact and reincarceration differed as a function of parole status. Among those who were not on parole after release, substance use treatment service contact was associated with an almost four-fold increase in the risk of reincarceration, and this effect persisted after adjustment for confounding. By contrast, among those released on parole there was no adjusted association between substance use treatment service contact and reincarceration. One interpretation of these findings is that parole facilitates earlier substance use treatment service access following release, which in turn attenuates the positive association between service use and reincarceration. Consistent with this, the average time to first contact with substance use treatment services was around three months earlier for those released on parole.

However, the vast majority of substance use treatment service users had only one (50%) or two (34%) service contacts during follow-up, and it seems unlikely that this level of treatment would be sufficient to substantively alter substance use trajectories in this population. Furthermore, our secondary analyses revealed that, for those not on parole, the effect of substance use treatment on reincarceration did not vary as a function of either the timing or the ‘dosage’ of service contact, suggesting that neither early engagement nor retention in substance use treatment was comparatively protective. Perhaps a more parsimonious explanation for the observed findings is that the degree of residual confounding of the effect of substance use service contact differed according to parole status: for those released on parole, engagement with substance use treatment services may have been mandated, such that it did not necessarily reflect current need for substance use treatment. By contrast, for those released to freedom (not on parole), substance use treatment service contact was likely in response to a recurrence of substance-related problems that we were unable to adequately control for in this study. As such, given our finding that late and infrequent engagement with behavioural health services was associated with increased risk of reincarceration, earlier and more frequent engagement with behavioural health services among this population may result in improved outcomes. This is an important area for future research.

### Strengths and limitations

This study is the first, to our knowledge, to prospectively investigate the association between use of community-based mental health and substance use treatment services following release from prison, and return to custody. The study is unique in that we considered mainstream, community-based services, rather than time-limited services targeting a particular subset of people released from prison. Other strengths of the study include the large and reasonably representative sample, combination of rich baseline survey data with seven linked administrative datasets, extensive adjustment for both baseline and time-varying confounders, and analysis of the effects of timing of and retention in treatment in secondary analyses.

Our study has several limitations. First, we were unable to disaggregate by treatment type (e.g., counselling *vs*. medication), such that it is unclear to what extent our findings would generalise to settings with a different mix of community-based mental health and substance use treatments. More targeted research is required to determine precisely what sorts of services warrant greater investment, although given the high prevalence of dual diagnosis in this population [37, 38], integrated substance use and mental health treatment, involving both counselling and pharmacotherapy, is likely pivotal. Second, we did not distinguish between reincarceration due to new offences and breaches of parole conditions, although we controlled for parole status in all models. As such, we cannot exclude the possibility that the relationship between behavioural health services and reincarceration due to parole breach and a new offence may differ. Future studies using population-level data linkage may have the capacity to explore this in greater detail. Third, despite extensive adjustment for both baseline and time-varying covariates, there is likely to be residual confounding in our study. In particular, we controlled only for lifetime indicators of substance use at baseline, which may not have been adequate to fully capture substance use problems at the time of release from custody. Further, our indicators of emergent mental health and substance use problems in the community relied on potentially incomplete administrative data, and our use of a thirty-day window to capture the time-varying nature of these effects was somewhat arbitrary. Fourth, although parole status can change after release from prison, we treated parole as a fixed covariate based on status at release. Fifth, our data are almost a decade old, although we are not aware of any substantive changes in the delivery of behavioural health services for people released from prisons in the state of Queensland during this time. Our cohort remains the largest and richest prospective cohort study of health outcomes for people released from prisons, anywhere in the world. However, there is a clear need for replication and extension of our findings in this and other jurisdictions. Finally, our findings point to an urgent need for further randomised trials of integrated behavioural health services for people released from prison, ideally stratifying by dual diagnosis status.

### Conclusions

In this large, prospective study we found that contact with mental health and substance use treatment services after release from prison was independently associated with increased risk of return to custody. Although we cannot eliminate the possibility of residual confounding, our careful and extensive adjustment for confounding suggests that delayed and inadequate engagement with behavioural health services may be not only inadequate to prevent reincarceration, but actually iatrogenic, challenging the widely-held view that such services are necessarily protective. Our findings provide strong evidence that infrequent or unsustained contact with community mental health and substance use treatment services after release from prison is insufficient to prevent reincarceration. There remains a pressing need for greater investment in the quality and timeliness of these services, and in systems for ensuring a smooth transition from prison-based to community care.

## Supporting information

S1 Table Definition of baseline variables. (DOCX)

S2 Table Definition of acute mental health and AOD service use variables. (DOCX)

S3 Table Treatments or services provided to those who contacted behavioural health services at least once. (DOCX)

S4 Table Full results from Model 2 and 3 Cox proportional hazards models for mental health service use and other variables prediction return to custody (N = 1,115). (DOCX)

S5 Table Full results from Model 2 and 3 Cox proportional hazards models for alcohol and drug service and other variables predicting return to custody, stratified by supervision status. (DOCX)

S6 Table Effect of behavioural health services on hazard of re-incarceration by timing of service initiation (N = 1,115). (DOCX)

S7 Table Effect of behavioural health services on hazard of re-incarceration by frequency of service contacts (N = 1,115). (DOCX)

S8 Table Effect of mental health and substance use services on hazard of re-incarceration, restricted to the control arm of the Passports intervention. (DOCX)

S1 Text Detailed methods for Model 3: Estimation of inverse probability of treatment weights. (DOCX)

## References

[1] Walmsley R. World prison population list (12th ed.). London: International Centre for Criminal Policy Research; 2018.

[2] Fazel S, Baillargeon J. The health of prisoners. Lancet. 2011;377(9769):956–65. doi: 10.1016/S0140-6736(10)61053-7 21093904

[3] Fazel S, Hayes AJ, Bartellas K, Clerici M, Trestman R. Mental health of prisoners: prevalence, adverse outcomes, and interventions. The Lancet Psychiatry. 2016;3(9):871–81. doi: 10.1016/S2215-0366(16)30142-0 27426440 PMC5008459

[4] Kinner SA, Milloy MJ, Wood E, Qi J, Zhang R, Kerr T. Incidence and risk factors for non-fatal overdose among a cohort of recently incarcerated illicit drug users. Addict Behav. 2012;37(6):691–6. doi: 10.1016/j.addbeh.2012.01.019 22385733 PMC3614083

[5] Winter RJ, Stoove M, Degenhardt L, Hellard ME, Spelman T, Jenkinson R, et al. Incidence and predictors of non-fatal drug overdose after release from prison among people who inject drugs in Queensland, Australia. Drug Alcohol Depend. 2015;153:43–9. doi: 10.1016/j.drugalcdep.2015.06.011 26105708

[6] Borschmann R, Thomas E, Moran P, Carroll M, Heffernan E, Spittal MJ, et al. Self-harm following release from prison: A prospective data linkage study. Aust N Z J Psychiatry. 2017;51(3):250–9. doi: 10.1177/0004867416640090 27012967

[7] Gan WQ, Kinner SA, Nicholls TL, Xavier CG, Urbanoski K, Greiner L, et al. Risk of overdose-related death for people with a history of incarceration. Addiction. 2021;116(6):1460–71. doi: 10.1111/add.15293 33047844

[8] Spittal MJ, Forsyth S, Pirkis J, Alati R, Kinner SA. Suicide in adults released from prison in Queensland, Australia: a cohort study. J Epidemiol Community Health. 2014;68(10):993–8. doi: 10.1136/jech-2014-204295 25009152

[9] Keen C, Kinner SA, Young JT, Jang K, Gan W, Samji H, et al. Prevalence of co-occurring mental illness and substance use disorder and association with overdose: a linked data cohort study among residents of British Columbia, Canada. Addiction. 2021. 10.1111/add.15580 34033179

[10] Kinner SA, Gan W, Slaunwhite A. High Rate of Fatal Overdose After Release from Prison In BC, Canada: A Data Linkage Study. International Journal of Population Data Science. 2020;5(5).

[11] Yukhnenko D, Sridhar S, Fazel S. A systematic review of criminal recidivism rates worldwide: 3-year update. Wellcome Open Res. 2019;4:28. doi: 10.12688/wellcomeopenres.14970.3 31544154 PMC6743246

[12] Hakansson A, Berglund M. Risk factors for criminal recidivism—a prospective follow-up study in prisoners with substance abuse. BMC Psychiatry. 2012;12:111. doi: 10.1186/1471-244X-12-111 22894706 PMC3492081

[13] Fazel S, Yu R. Psychotic Disorders and Repeat Offending: Systematic Review and Meta-analysis. Schizophrenia Bulletin. 2011;37(4):800–10. doi: 10.1093/schbul/sbp135 19959703 PMC3122287

[14] Thomas EG, Spittal MJ, Taxman FS, Kinner SA. Health-related factors predict return to custody in a large cohort of ex-prisoners: new approaches to predicting re-incarceration. Health & Justice. 2015;3(1).

[15] Rojas EC, Peters RH. Evidence-based practices for co-occurring disorders in offenders. Addiction Research & Theory. 2016;24(3):223–35.

[16] Held ML, Brown CA, Frost LE, Hickey JS, Buck DS. Integrated Primary and Behavioral Health Care in Patient-Centered Medical Homes for Jail Releasees With Mental Illness. Criminal Justice and Behavior. 2012;39:533–51.

[17] Hsieh M-L, Hamilton ZK. Predicting Success in Residential Substance Abuse Interventions: New Jerseys Pre-Release Incarceration Alternatives. Criminal Justice Policy Review. 2016;27:182–202.

[18] Domino ME, Gertner A, Grabert B, Cuddeback GS, Childers T, Morrissey JP. Do timely mental health services reduce re-incarceration among prison releasees with severe mental illness? Health Serv Res. 2019;54(3):592–602. doi: 10.1111/1475-6773.13128 30829406 PMC6505414

[19] Draine J, Solomon P. Describing and evaluating jail diversion services for persons with serious mental illness. Psychiatr Serv. 1999;50(1):56–61. doi: 10.1176/ps.50.1.56 9890580

[20] Draine J, Solomon P. Threats of incarceration in a psychiatric probation and parole service. Am J Orthopsychiatry. 2001;71(2):262–7. doi: 10.1037/0002-9432.71.2.262 11347368

[21] Solomon P, Draine J. One-Year Outcomes of a Randomized Trial of Case Management with Seriously Mentally Ill Clients Leaving Jail. Evaluation Review. 2016;19(3):256–73.

[22] Solomon P, Draine J, Marcus SC. Predicting incarceration of clients of a psychiatric probation and parole service. Psychiatr Serv. 2002;53(1):50–6. doi: 10.1176/appi.ps.53.1.50 11773649

[23] Kinner SA, Lennox N, Williams GW, Carroll M, Quinn B, Boyle F, et al. Randomised controlled trial of a service brokerage intervention for ex-prisoners in Australia. Contemporary Clinical Trails. 2013;36:198–206. doi: 10.1016/j.cct.2013.07.001 23850859

[24] Larney S, Burns L. Evaluating health outcomes of criminal justice populations using record linkage: the importance of aliases. Evaluation Review. 2011;35(2):118–28. doi: 10.1177/0193841X11401695 21398273

[25] Lawrence G, Dinh I, Taylor L. The Centre for Health Record Linkage: A New Resource for Health Services Research and Evaluation. Health Inf Manag. 2008;37(2):60–2. doi: 10.1177/183335830803700208 28758494

[26] Young JT, van Dooren K, Lennox NG, Butler TG, Kinner SA. Inter-rater reliability of the Hayes Ability Screening Index in a sample of Australian prisoners. J Intellect Disabil Res. 2015;59(11):1055–60. doi: 10.1111/jir.12198 26018331

[27] Kessler RC, Andrews G, Colpe LJ, Hiripi E, Mroczek DK, Normand S-L, et al. Short screening scales to monitor population prevalences and trends in non-specific psychological distress. Psychological medicine. 2002;32(06):959–76. doi: 10.1017/s0033291702006074 12214795

[28] Ware JE, Kosinski M, Dewey JE. How to score version 2 of the SF-36 health survey (standard & acute forms): QualityMetric Incorporated; 2000.

[29] Kinner SA, Jenkinson R, Gouillou M, Milloy MJ. High-risk drug-use practices among a large sample of Australian prisoners. Drug Alcohol Depend. 2012;126(1–2):156–60. doi: 10.1016/j.drugalcdep.2012.05.008 22658284

[30] Babor TF, Higgins-Biddle JC, Saunders JB, Monteiro MG. The Alcohol Use Disorders Identification Test: Guidelines for Use in Primary Care. Geneva. Geneva: World Health Organization, Department of Mental Health and Substance Dependence; 2001.

[31] Humeniuk R, Henry-Edwards S, Ali R, Poznyak V, Monteiro MG. The Alcohol, Smoking and Substance Involvement Screening Test (ASSIST): manual for use in primary care. Geneva: World Health Organisation; 2010. Report No.: 9522727083.

[32] Robins JM, Hernan MA, Brumback B. Marginal structural models and causal inference in epidemiology. Epidemiology. 2000;11(5):550–60. doi: 10.1097/00001648-200009000-00011 10955408

[33] Xiao Y, Abrahamowicz M, Moodie EE. Accuracy of conventional and marginal structural Cox model estimators: a simulation study. The International Journal of Biostatistics. 2010;6(2). doi: 10.2202/1557-4679.1208 21969997

[34] Keen C, Kinner SA, Young JT, Snow K, Zhao B, Gan W, et al. Periods of altered risk for non-fatal drug overdose: a self-controlled case series. The Lancet Public Health. 2021;6(4):e249–e59. doi: 10.1016/S2468-2667(21)00007-4 33773635

[35] Young JT, Arnold-Reed D, Preen D, Bulsara M, Lennox N, Kinner SA. Early primary care physician contact and health service utilisation in a large sample of recently released ex-prisoners in Australia: prospective cohort study. BMJ Open. 2015;5(e6). 10.1136/bmjopen-2015-008021 PMC4466622 26068513

[36] Thomas EG, Spittal MJ, Heffernan EB, Taxman FS, Alati R, Kinner SA. Trajectories of psychological distress after prison release: implications for mental health service need in ex-prisoners. Psychol Med. 2016;46(3):611–21. doi: 10.1017/S0033291715002123 26549475

[37] Borschmann R, Dos Santos MM, Young JT, Andreoli SB, Love AD, Kinner SA. Health, social and criminal justice factors associated with dual diagnosis among incarcerated adults in Brazil and Australia: a cross-national comparison. Soc Psychiatry Psychiatr Epidemiol. 2020;55(10):1355–62. doi: 10.1007/s00127-020-01832-w 32047971

[38] Kinner SA, Borschmann R. Dual-harm, complex needs, and the challenges of multisectoral service coordination. The Lancet Public Health. 2019;4(5):e210–e1. doi: 10.1016/S2468-2667(19)30065-9 31054634

