# Supplementary material for: Association between contact with mental health and substance use services and reincarceration after release from prison
Source: PLoS One. 2022 Sep 7;17(9):e0272870. doi: 10.1371/journal.pone.0272870 (PMC9451082; doi:10.1371/journal.pone.0272870)
Supplement: S1 Table — (DOCX) [file pone.0272870.s001.docx]

**Table S1:** Definition of baseline variables

| **Variable** | **Definition^a^ (Number with missing data of 1,307 participants linked to all datasets)** |
| --- | --- |
| **Demographics** |  |
| Age | Age on day of release from index incarceration. (0) |
| Female | Female gender. (0) |
| Indigenous | Australian Aboriginal and/or Torres Strait Islander. (0) |
| Not married or de-facto | Not married or in a de-facto relationship at time of baseline interview. (0) |
| **Criminal justice** |  |
| Prior adult incarceration | Any prior incarcerations aged ≥17 years according to correctional records. (2) |
| Juvenile incarceration | Any incarcerations aged <17 years. (13) |
| Drug-related sentence | Drug related sentence according to correctional records. (8) |
| Violent offence | Most serious offence leading to index incarceration was violent (including sexual offences) according to the Australian Standard Offence Classification Queensland Supplement [1]. (8) |
| ROR Score | Risk of reoffending score used by Queensland Corrective Services. (11) |
| **Lifetime mental health problems** |  |
| Anxiety disorder, mood disorder and schizophrenia | Responded yes to: “Have you ever been told by a doctor, psychologist or psychiatrist that you have a mental illness?”, and specified one of the diagnoses at left. These questions were adapted from Australia’s National Health Survey [2]. (Number with missing data: 8 for anxiety, 6 for mood disorder, 3 for schizophrenia) |
| **Mental health problems at baseline** |  |
| CNS medications | Central Nervous System (CNS) medication (defined according to MIMS) [3] prescription at time of baseline interview, according to QCS health records accessed with participant consent. (94) |
| Screens positive for intellectual disability | At least two of: scored <84.5 on the Hayes Ability Screening Index (HASI) [4]; attended a special school; has been diagnosed with an intellectual disability (22) |
| Kessler Psychological Distress Scale (K10) | A validated survey instrument measuring non-specific psychological distress [5]. Scores ≥22 indicate high or very high distress. (5) |
| SF-36 mental component summary score (MCSAT) | A validated survey instrument measuring mental health-related functioning [6]. Australian T-normed scores were used [7]. (3) |
| **Lifetime Alcohol and other Drug (AOD) problems** |  |
| Ever injected illicit drugs | Any previous injecting use of illicit drugs. (3) |
| Ever shared injecting equipment | Responded yes to at least one of the following questions: “Have you ever used a needle after someone else had already used it?”, or; “Have you ever used other injecting equipment (e.g., water, spoon, tourniquet) after someone else had already used it?” (3) |
| Ever injected in prison | Ever injected illicit drugs while incarcerated. (3) |
| Ever overdosed | Responded yes to: “Have you ever overdosed or become unconscious as a result of taking drugs?” (8) |
| Pre-prison AOD problems |  |
| Alcohol Use Disorders Identification Test (AUDIT) | A validated survey instrument measuring risk of alcohol-related harm [8]. Participants were asked to respond with reference to their drinking in the six months prior to incarceration. A score ≥16 indicates high risk to possibly dependent drinking. (29) |
| Alcohol Smoking and Substance Involvement Screening Test (ASSIST) | A validated survey instrument measuring risk of harm due to illicit drug use [9]. Responses are given for a range of substances. We considered scores for cannabis, amphetamines, heroin and other opioids such as morphine (3). Participants were asked to respond with reference to their drug use in the three months prior to incarceration. Scores ≥4 indicate risky use of that substance. (Number with missing data: 1 for cannabis, 2 for amphetamines, 3 for heroin) |
| Social support |  |
| No visits past four weeks | Not visited in prison by any community contacts in the four weeks prior to baseline interview. (0) |
| ENrICHD Social Support Inventory (ESSI) | A validated survey instrument measuring perceived social support [10]. Scores ≤2 on at least two of five items and a total score of ≤18 indicate low perceived social support [11]. (3) |
| **Socioeconomic status (SES)** |  |
| <10 years education | Less than 10 years of school attended. (4) |
| Below poverty line | Income in 4 weeks before incarceration below poverty line according to a published Australian standard [12], accounting for dependents and marital status. (2) |
| Unstable housing | No stable accommodation in the month prior to incarceration. (0) |
| Unemployed | No part-time, full-time or casual employment in the 6 months prior to incarceration (0) |
| **Other** |  |
| Post-release postcode | Participant’s most likely residential postcode after return to the community, categorized by remoteness area (major city, inner/outer regional or remote/very remote) according to the Australian Statistical Geography Standard [13]. For postcodes split between multiple remoteness areas, we used the remoteness area representing the greatest percentage of that postcode. (21) |
| IX arm, Passports | Intervention arm of the Passports study [14]. (0) |

^a^Variables are from participant self-report unless otherwise stated.

1. OESR. Australian standard offence classification (Queensland extension). Brisbane, Australia: Office of Economic and Statistical Research, Queensland Government; 2008.

2. ABS. National Health Survey: users' guide - electronic. Canberra: Australian Bureau of Statistics; 2008. Contract No.: 4363.0.55.001.

3. MIMS Online [Internet]. CMPMedica Australia Pty Ltd. Available from: <www.mimsonline.com.au>.

4. Hayes SC. Hayes ability screening index (HASI) manual. Faculty of Medicine, University of Sydney; 2000.

5. Kessler RC, Andrews G, Colpe LJ, Hiripi E, Mroczek DK, Normand S-L, et al. Short screening scales to monitor population prevalences and trends in non-specific psychological distress. Psychological medicine. 2002;32(06):959-76.

6. Ware JE, Kosinski M, Dewey JE. How to score version 2 of the SF-36 health survey (standard & acute forms): QualityMetric Incorporated; 2000.

7. ABS. National Health Survey: SF-36 population norms, Australia. Canberra: Australian Bureau of Statistics; 1997.

8. Babor TF, Higgins-Biddle JC, Saunders JB, Monteiro MG. The Alcohol Use Disorders Identification Test: Guidelines for Use in Primary Care. Geneva. Geneva: World Health Organization, Department of Mental Health and Substance Dependence; 2001.

9. Humeniuk R, Henry-Edwards S, Ali R, Poznyak V, Monteiro MG. The Alcohol, Smoking and Substance Involvement Screening Test (ASSIST): manual for use in primary care. Geneva: World Health Organisation; 2010. Report No.: 9522727083.

10. Mitchell PH, Powell L, Blumenthal J, Norten J, Ironson G, Pitula CR, et al. A short social support measure for patients recovering from myocardial infarction: the ENRICHD social support inventory. Journal of Cardiopulmonary Rehabilitation and Prevention. 2003;23(6):398-403.

11. ENrICHD Investigators. Enhancing recovery in coronary heart disease (ENRICHD): baseline characteristics. The American Journal of Cardiology. 2001;88(3):316-22.

12. MIAESR. Poverty lines: Australia Melbourne: Melbourne Institute of Applied Economic and Social Research, University of Melbourne; 2014 [

13. ABS. Australian Statistical Geography Standard (ASGS): correspondences, July 2011. Canberra, Australia: Australian Bureau of Statistics; 2012. Contract No.: 1270.0.55.006.

14. Kinner SA, Lennox N, Williams GW, Carroll M, Quinn B, Boyle F, et al. Randomised controlled trial of a service brokerage intervention for ex-prisoners in Australia. Contemporary Clinical Trails. 2013;36:198-206.
