# Supplementary material for: Association between contact with mental health and substance use services and reincarceration after release from prison
Source: PLoS One. 2022 Sep 7;17(9):e0272870. doi: 10.1371/journal.pone.0272870 (PMC9451082; doi:10.1371/journal.pone.0272870)
Supplement: S2 Table — (DOCX) [file pone.0272870.s002.docx]

### **Table S2.** Definition of acute mental health and AOD service use variables

| **Variable** | **Definition** |
| --- | --- |
| Hospital admissions |  |
| Mental health-related | Primary diagnosis fell under mental and behavioural disorders, excluding those due to psychoactive substances (all ICD-10^a^ codes beginning with F, excluding F10-F19), as well as any episodes in which the patient was admitted for psychiatric treatment. |
| AOD-related | Primary diagnosis fell under mental and behaviour disorders due to psychoactive substance (ICD-10^a^ codes F10-F19). |
| Emergency department presentations | |
| Mental health-related | Primary diagnosis fell under mental and behavioural disorders, excluding those due to psychoactive substances (all ICD-10^a^ codes beginning with F, excluding F10-F19). |
| AOD-related | Primary diagnosis fell under mental and behaviour disorders due to psychoactive substance (ICD-10^a^ codes F10-F19). |
| Ambulance callouts |  |
| Mental health-related | Case definition recorded by paramedics as psychiatric or emotional problem. |
| AOD-related | Case definition recorded by paramedics as alcohol/drug withdrawal, overdose/exposure or alcohol/drug requesting detox. |

^a^International Statistical Classification of Diseases and Related Health Problems, 10^th^ Revision (WHO, 2015)
