# Supplementary material for: Association between contact with mental health and substance use services and reincarceration after release from prison
Source: PLoS One. 2022 Sep 7;17(9):e0272870. doi: 10.1371/journal.pone.0272870 (PMC9451082; doi:10.1371/journal.pone.0272870)
Supplement: S3 Table — (DOCX) [file pone.0272870.s003.docx]

**Table S3:** Treatments or services provided to those who contacted behavioural health services at least once

| **Mental health services (n=215)** | | **Alcohol and other drug services (n=270)** | |
| --- | --- | --- | --- |
| **Type of treatment or service provided** | **Percent (number)** | **Type of treatment or service provided** | **Percent (number)** |
| Assessment | 54.4 (116) | Assessment | 35.6 (96) |
| Intake | 57.7 (124) | Counselling | 34.1 (92) |
| Counselling/therapy | 19.5 (42) | Pharmacotherapy | 20.7 (56) |
| Crisis management | 20.5 (44) | Rehabilitation | 1.1 (3) |
| Case management | 7.9 (17) | Case management | 5.9 (16) |
| Review | 14.0 (30) | Withdrawal management | 7.0 (19) |
| Education | 7.0 (15) | Other | 2.6 (7) |
| Reporting/documentation | 13.5 (29) |  |  |
| Second opinion | 6.5 (14) |  |  |
| Other | 39.5 (85) |  |  |

The table shows the percentage of participants who had at least one behavioural health service contact (mental health or substance use services) of each type, among those participants who had at least one service contact before return to custody, death or censoring. Note that the service types are not mutually exclusive (hence the percentages do not add to 100%), in that a single participant may have received multiple service types during the study period.
