# Supplementary material for: Association between contact with mental health and substance use services and reincarceration after release from prison
Source: PLoS One. 2022 Sep 7;17(9):e0272870. doi: 10.1371/journal.pone.0272870 (PMC9451082; doi:10.1371/journal.pone.0272870)
Supplement: S4 Table — (DOCX) [file pone.0272870.s004.docx]

**Table S4:** Full results from Model 2 and 3 Cox proportional hazards models for mental health service use and other variables prediction return to custody (N=1,115)

| **Variable** | **Model 2** | **Model 3**^a^ |
| --- | --- | --- |
| Mental health services | 1.97 (1.43, 2.71) | 1.76 (1.23, 2.51) |
| Age^b^ | 0.80 (0.71, 0.91) | 0.79 (0.69, 0.90) |
| Female | 0.70 (0.54, 0.91) | 0.77 (0.59, 1.01) |
| Indigenous | 1.24 (0.97, 1.57) | 1.19 (0.92, 1.53) |
| Not married or de-facto | 1.00 (0.82, 1.23) | 1.08 (0.87, 1.33) |
| Prior incarcerations (adult) | 1.68 (1.26, 2.23) | 1.79 (1.33, 2.41) |
| Juvenile incarcerations | 1.52 (1.24, 1.87) | 1.51 (1.22, 1.87) |
| Violent offence | 1.12 (0.93, 1.37) | 1.14 (0.93, 1.40) |
| Drug-related sentence | 1.23 (1.00, 1.51) | 1.27 (1.02, 1.57) |
| ROR score | 1.05 (1.03, 1.07) | 1.05 (1.03, 1.08) |
| <10 years education | 0.92 (0.75, 1.11) | 0.94 (0.76, 1.15) |
| Unstable housing^c^ | 1.03 (0.83, 1.29) | 0.97 (0.76, 1.25) |
| Unemployed^c^ | 1.08 (0.88, 1.33) | 1.02 (0.82, 1.26) |
| Below poverty line^c^ | 1.14 (0.93, 1.38) | 1.15 (0.93, 1.41) |
| Post-release postcode |  |  |
| Regional | 0.82 (0.65, 1.03) | 0.88 (0.69, 1.12) |
| Remote | 0.79 (0.45, 1.41) | 0.84 (0.46, 1.52) |
| K10 score^b^ | 1.01 (0.83, 1.22) | 0.94 (0.77, 1.15) |
| ESSI score^b^ | 0.94 (0.80, 1.09) | 0.95 (0.80, 1.11) |
| No visits past four weeks | 1.08 (0.89, 1.32) | 1.09 (0.89, 1.33) |
| CNS medications | 1.17 (0.91, 1.49) | 1.32 (1.03, 1.69) |
| Mood disorder | 1.20 (0.94, 1.54) | 1.22 (0.95, 1.57) |
| Anxiety disorder | 0.93 (0.62, 1.39) | 0.93 (0.62, 1.42) |
| Schizophrenia | 0.73 (0.44, 1.22) | 0.56 (0.31, 1.03) |
| SF-36 MCSAT^b^ | 1.13 (1.01, 1.26) | 1.08 (0.96, 1.22) |
| Overdose | 0.89 (0.70, 1.12) | 0.90 (0.70, 1.16) |
| Shared injecting equipment | 1.19 (0.92, 1.54) | 1.26 (0.96, 1.66) |
| Injected in prison | 0.99 (0.75, 1.30) | 0.98 (0.73, 1.31) |
| Injecting drug use | 1.21 (0.94, 1.55) | 1.22 (0.95, 1.58) |
| AUDIT score^b^ | 1.08 (0.98, 1.19) | 1.05 (0.95, 1.17) |
| ASSIST score (cannabis)^b^ | 1.00 (0.92, 1.09) | 0.99 (0.90, 1.08) |
| ASSIST score (heroin)^b^ | 1.16 (1.05, 1.28) | 1.14 (1.03, 1.26) |
| ASSIST score (other opioids)^b^ | 0.82 (0.71, 0.94) | 0.82 (0.71, 0.94) |
| ASSIST score (methamphetamine)^b^ | 1.13 (1.04, 1.23) | 1.13 (1.03, 1.23) |
| IX arm, Passports | 1.03 (0.86, 1.22) | 1.04 (0.86, 1.25) |

^a^Further adjusted for time-varying covariates using inverse probability of treatment weighting

^b^HRs for all continuous variables refer to ten point increments of that variable

^c^Prior to index incarceration
