# Supplementary material for: Association between contact with mental health and substance use services and reincarceration after release from prison
Source: PLoS One. 2022 Sep 7;17(9):e0272870. doi: 10.1371/journal.pone.0272870 (PMC9451082; doi:10.1371/journal.pone.0272870)
Supplement: S5 Table — (DOCX) [file pone.0272870.s005.docx]

**Table S5:** Full results from Model 2 and 3 Cox proportional hazards models for alcohol and drug service and other variables predicting return to custody, stratified by supervision status

| **Variable** | **Not under supervision (n = 442)** | | **Under supervision (n = 673)** | |
| --- | --- | --- | --- | --- |
|  | **Model 2** | **Model 3**^a^ | **Model 2** | **Model 3**^a^ |
| AOD services | 2.99 (2.03, 4.40) | 3.16 (2.09, 4.78) | 1.10 (0.82, 1.46) | 1.07 (0.80, 1.43) |
| Age^b^ | 0.70 (0.55, 0.89) | 0.71 (0.55, 0.90) | 0.85 (0.73, 0.99) | 0.84 (0.72, 0.98) |
| Female | 0.60 (0.36, 1.00) | 0.61 (0.36, 1.03) | 0.64 (0.48, 0.86) | 0.66 (0.49, 0.89) |
| Indigenous | 1.05 (0.63, 1.73) | 1.03 (0.62, 1.70) | 1.29 (0.98, 1.70) | 1.27 (0.96, 1.68) |
| Not married or de-facto | 0.94 (0.64, 1.39) | 0.92 (0.61, 1.37) | 1.14 (0.88, 1.46) | 1.14 (0.89, 1.46) |
| Prior incarcerations (adult) | 2.16 (1.24, 3.75) | 2.17 (1.26, 3.74) | 1.60 (1.13, 2.28) | 1.63 (1.14, 2.32) |
| Juvenile incarcerations | 1.65 (1.11, 2.44) | 1.60 (1.07, 2.39) | 1.57 (1.22, 2.02) | 1.60 (1.24, 2.06) |
| Violent offence | 0.94 (0.67, 1.33) | 0.92 (0.64, 1.31) | 1.13 (0.90, 1.41) | 1.12 (0.89, 1.41) |
| Drug-related sentence | 1.50 (1.05, 2.14) | 1.51 (1.05, 2.17) | 1.12 (0.88, 1.43) | 1.15 (0.90, 1.46) |
| ROR Score | 1.04 (1.00, 1.08) | 1.04 (1.00, 1.08) | 1.06 (1.03, 1.09) | 1.06 (1.03, 1.09) |
| <10 years education | 0.87 (0.62, 1.23) | 0.92 (0.64, 1.31) | 0.89 (0.70, 1.13) | 0.88 (0.69, 1.11) |
| Unstable housing^c^ | 1.14 (0.77, 1.67) | 1.00 (0.66, 1.50) | 1.09 (0.83, 1.42) | 1.09 (0.83, 1.42) |
| Unemployed^c^ | 1.07 (0.73, 1.57) | 1.11 (0.75, 1.64) | 1.18 (0.92, 1.51) | 1.19 (0.93, 1.53) |
| Below poverty line^c^ | 1.48 (1.03, 2.15) | 1.43 (0.99, 2.07) | 1.05 (0.82, 1.34) | 1.05 (0.82, 1.34) |
| Post-release postcode |  |  | (, ) |  |
| Regional | 0.98 (0.65, 1.48) | 0.95 (0.62, 1.45) | 0.81 (0.61, 1.07) | 0.81 (0.61, 1.08) |
| Remote | 0.50 (0.12, 1.99) | 0.42 (0.09, 1.91) | 0.89 (0.46, 1.70) | 0.91 (0.47, 1.73) |
| K10 score^b^ | 1.25 (0.85, 1.83) | 1.36 (0.90, 2.06) | 0.94 (0.74, 1.21) | 0.92 (0.71, 1.18) |
| ESSI score^b^ | 1.06 (0.81, 1.40) | 0.94 (0.70, 1.26) | 0.76 (0.63, 0.91) | 0.76 (0.63, 0.91) |
| No visits past four weeks | 1.08 (0.74, 1.58) | 0.97 (0.66, 1.42) | 1.14 (0.90, 1.44) | 1.13 (0.89, 1.44) |
| CNS medications | 1.00 (0.66, 1.52) | 0.99 (0.64, 1.52) | 1.61 (1.22, 2.13) | 1.60 (1.21, 2.12) |
| Mood disorder | 1.30 (0.83, 2.04) | 1.45 (0.92, 2.29) | 1.00 (0.73, 1.37) | 1.00 (0.73, 1.38) |
| Anxiety disorder | 0.67 (0.29, 1.58) | 0.65 (0.26, 1.62) | 1.18 (0.79, 1.77) | 1.26 (0.84, 1.90) |
| Schizophrenia | 0.96 (0.42, 2.19) | 0.78 (0.33, 1.84) | 1.12 (0.63, 1.99) | 1.01 (0.56, 1.82) |
| SF-36 MCSAT^b^ | 1.16 (0.92, 1.45) | 1.17 (0.93, 1.48) | 1.13 (0.98, 1.30) | 1.12 (0.98, 1.28) |
| Overdose | 1.07 (0.73, 1.57) | 1.08 (0.73, 1.59) | 0.81 (0.60, 1.09) | 0.81 (0.60, 1.10) |
| Shared injecting equipment | 0.92 (0.59, 1.45) | 0.91 (0.58, 1.44) | 1.45 (1.05, 2.01) | 1.48 (1.06, 2.05) |
| Injected in prison | 1.58 (0.99, 2.51) | 1.51 (0.94, 2.43) | 1.01 (0.72, 1.43) | 0.96 (0.68, 1.37) |
| Injecting drug use | 0.91 (0.54, 1.53) | 0.93 (0.55, 1.58) | 1.26 (0.92, 1.72) | 1.26 (0.93, 1.72) |
| AUDIT score^b^ | 1.13 (0.93, 1.37) | 1.16 (0.95, 1.41) | 1.02 (0.91, 1.15) | 1.03 (0.91, 1.16) |
| ASSIST score (cannabis)^b^ | 1.05 (0.89, 1.23) | 1.08 (0.91, 1.27) | 0.92 (0.83, 1.01) | 0.92 (0.83, 1.01) |
| ASSIST score (heroin)^b^ | 1.11 (0.92, 1.32) | 1.09 (0.89, 1.33) | 1.14 (1.00, 1.30) | 1.15 (1.01, 1.31) |
| ASSIST score (other opioids)^b^ | 0.72 (0.56, 0.94) | 0.74 (0.56, 0.98) | 0.86 (0.72, 1.02) | 0.88 (0.74, 1.04) |
| ASSIST score (methamphetamine)^b^ | 1.18 (1.02, 1.36) | 1.22 (1.04, 1.42) | 1.10 (0.99, 1.21) | 1.09 (0.99, 1.21) |
| IX arm, Passports | 1.45 (1.06, 2.00) | 1.33 (0.94, 1.86) | 0.93 (0.74, 1.15) | 0.91 (0.73, 1.14) |

^a^Further adjusted for time-varying covariates using inverse probability of treatment weighting

^b^HRs for all continuous variables refer to ten point increments of that variable

^c^Prior to index incarceration
