# Supplementary material for: Association between contact with mental health and substance use services and reincarceration after release from prison
Source: PLoS One. 2022 Sep 7;17(9):e0272870. doi: 10.1371/journal.pone.0272870 (PMC9451082; doi:10.1371/journal.pone.0272870)
Supplement: S6 Table — (DOCX) [file pone.0272870.s006.docx]

**Table S6:** Effect of behavioural health services on hazard of re-incarceration by timing of service initiation (N=1,115)

| Model | **Early initiators**  **HR (95%CI)** | **Late initiators**  **HR (95%CI)** | **P-value for difference** |
| --- | --- | --- | --- |
| Mental health services |  |  |  |
| Model 1^a^ | 1.76 (1.23, 2.52) | 3.19 (2.33, 4.36) | 0.011 |
| Model 2^b^ | 1.32 (0.86, 2.02) | 2.32 (1.59, 3.40) | 0.032 |
| Model 3^c^ | 1.09 (0.69, 1.73) | 2.15 (1.41, 3.29) | 0.020 |
| AOD services for unsupervised ex-prisoners |  |  |  |
| Model 1^a^ | 3.08 (1.82, 5.24) | 4.86 (3.25, 7.27) | 0.139 |
| Model 2^b^ | 2.11 (1.08, 4.12) | 3.46 (2.29, 5.20) | 0.173 |
| Model 3^c^ | 2.47 (1.11, 5.49) | 3.51 (2.28, 5.42) | 0.411 |

^a^Unadjusted

^b^Adjusted for pre-release covariates

^c^Adjusted for pre-release covariates and post-release (time-varying) covariates ‘

The table shows results from secondary analyses for our Cox proportional hazards models for time to re-incarceration. Early initiators are those who contacted services ≤30 days after release from index incarceration; late initiators first contacted services >30 days after release. For each model, we present the estimated hazard ratios (HRs) and 95% confidence intervals (CIs). The HRs for each primary exposure are also adjusted for the other primary exposure, such that the results shown for each phase are from one model only. The p-values shown are from Wald tests of the null hypothesis that the HRs for early and late initiators are equal.
