# Supplementary material for: Association between contact with mental health and substance use services and reincarceration after release from prison
Source: PLoS One. 2022 Sep 7;17(9):e0272870. doi: 10.1371/journal.pone.0272870 (PMC9451082; doi:10.1371/journal.pone.0272870)
Supplement: S7 Table — (DOCX) [file pone.0272870.s007.docx]

**Table S7:** Effect of behavioural health services on hazard of re-incarceration by frequency of service contacts (N=1,115)

| **Model** | **Single service users**  **HR (95%CI)** | **Multiple service users**  **HR (95%CI)** | **P-value for difference** |
| --- | --- | --- | --- |
| Mental health services |  |  |  |
| Model 1^a^ | 2.53 (1.78, 3.60) | 2.61 (1.86, 3.66) | 0.899 |
| Model 2^b^ | 1.86 (1.20, 2.90) | 2.09 (1.41, 3.07) | 0.684 |
| Model 3^c^ | 1.82 (1.12, 2.96) | 1.68 (1.09, 2.60) | 0.790 |
| AOD services for unsupervised ex-prisoners |  |  |  |
| Model 1^a^ | 4.00 (2.69, 5.93) | 4.67 (2.54, 8.58) | 0.654 |
| Model 2^b^ | 2.90 (1.93, 4.38) | 3.43 (1.68, 7.00) | 0.653 |
| Model 3^c^ | 3.11 (2.00, 4.83) | 3.41 (1.62, 7.19) | 0.814 |

^a^Unadjusted

^b^Adjusted for pre-release covariates

^c^Adjusted for pre-release covariates and post-release (time-varying) covariates

The table shows results from secondary analyses for our Cox proportional hazards models for time to re-incarceration. Single service users are those who contacted services exactly once during follow-up; multiple service users contacted services more than once. For each model, we present the estimated hazard ratios (HRs) and 95% confidence intervals (CIs). The HRs for each primary exposure are also adjusted for the other primary exposure, such that the results shown for each phase are from one model only. The p-values shown are from Wald tests of the null hypothesis that the HRs for single and multiple service users are equal.
