# Supplementary material for: Association between contact with mental health and substance use services and reincarceration after release from prison
Source: PLoS One. 2022 Sep 7;17(9):e0272870. doi: 10.1371/journal.pone.0272870 (PMC9451082; doi:10.1371/journal.pone.0272870)
Supplement: S8 Table — (DOCX) [file pone.0272870.s008.docx]

**Table S8:** Effect of mental health and substance use services on hazard of re-incarceration, restricted to the control arm of the Passports intervention

|  | **Mental health services** |  | **Substance use services** | |
| --- | --- | --- | --- | --- |
|  | **All**  **(n=553)** |  | **Not on parole**  **(n=207)** | **On parole**  **(n=346)** |
| **Model** | **HR (95%CI)** |  | **HR (95%CI)** | **HR (95%CI)** |
| Model 1^a^ | 2.18 (1.51, 3.16) |  | 3.50 (2.05, 5.98) | 1.67 (1.16, 2.41) |
| Model 2^b^ | 1.76 (1.14, 2.71) |  | 3.19 (1.76, 5.77) | 1.43 (0.95, 2.15) |
| Model 3^c^ | 1.63 (1.03, 2.59) |  | 2.94 (1.51, 5.70) | 1.38 (0.92, 2.09) |

^a^Unadjusted

^b^Adjusted for pre-release covariates

^c^Adjusted for pre-release covariates and post-release (time-varying) covariates
