# Supplementary material for: Association between contact with mental health and substance use services and reincarceration after release from prison
Source: PLoS One. 2022 Sep 7;17(9):e0272870. doi: 10.1371/journal.pone.0272870 (PMC9451082; doi:10.1371/journal.pone.0272870)
Supplement: S1 Text — (DOCX) [file pone.0272870.s009.docx]

**Supplementary Text 1.** Detailed methods for Model 3: estimation of inverse probability of treatment weights

In Model 3, we used inverse-probability-of-treatment weights (IPTWs) to fit a marginal structural model (MSM) controlling for potential time-varying confounders. MSMs can be used to control for time-varying confounders that both predict future exposure and lie on the causal pathway between past exposure and the outcome (Robins et al., 2000). In our study, we hypothesised that need for acute mental health/AOD treatment would increase the chance of both future behavioural health service use and return to custody, and is hence a confounder of the relationship between exposure and outcome. However, behavioural health service access may also reduce future need for acute mental health/AOD-related treatment, in turn reducing the risk of return to custody, such that acute mental health/AOD-related treatment also lies on the causal pathway between exposure and outcome. In situations such as this, traditional adjustment using time-varying covariates in survival analysis may bias the estimate of the causal effect of exposure on outcome. Inverse probability of treatment weighting attempts to solve this problem by constructing, for each risk set in the Cox model, a weighted pseudo population in which the time-varying confounder is no longer associated with the exposure.

To calculate the IPTWs, we followed the approach described by Hernán et al (2000). For each exposure of interest (contact with community mental health and AOD services), we ran pooled logistic regression models in which the unit of analysis was each person-day in the study, and the exposure variable was treated as a binary outcome. All time-varying and baseline covariates were included in the models used to calculate the denominator of the standardised weights, and only the baseline covariates were included in the models used to calculate the numerator. For all models, we accounted for the effect of time since release using natural cubic splines with five internal knots at the 5^th^, 25^th^, 50^th^, 75^th^ and 95^th^ percentiles of the time variable. We did not estimate censoring weights since censoring in our study was uninformative – all participants were censored on the essentially arbitrary date of linkage for the correctional data, such that time until censoring was unlikely to be associated with any variables included in our models. Estimation of the IPTWs was performed in Stata version 13.1, following the approach described by Fewell et al (2004).
